# Risk of Severe Adverse Maternal and Neonatal Outcomes in Deliveries with Repeated and Primary Cesarean Deliveries versus Vaginal Deliveries: A Cross-Sectional Study

**DOI:** 10.1155/2020/9207431

**Published:** 2020-05-04

**Authors:** Kiattisak Kongwattanakul, Rungroj Thamprayoch, Chumnan Kietpeerakool, Pisake Lumbiganon

**Affiliations:** Department of Obstetrics and Gynecology, Faculty of Medicine, Khon Kaen University, 40002, Thailand

## Abstract

**Objective:**

To determine risks of severe adverse maternal and neonatal outcomes in women with repeated cesarean delivery (CD) and primary CD compared with those with vaginal delivery (VD).

**Methods:**

Data of this cross-sectional study were extracted from 2,262 pregnant women who gave birth between August 2014 and December 2016, at Srinagarind Hospital, Khon Kaen University. Severe maternal outcomes were categorized based on the World Health Organization criteria. Adjusted odds ratio (aOR) and 95% confidence intervals (CI) were calculated to indicate the risk of severe adverse maternal and neonatal outcomes among women underwent CD compared with those who underwent VD.

**Results:**

There were no cases of maternal death in this study. CD significantly increased risk of severe adverse maternal outcomes (SMO) (aOR 10.59; 95% CI, 1.19-94.54 for primary CD and aOR 17.21; 95% CI, 1.97-150.51 for repeated CD) compared with women who delivered vaginally. When compared with vaginal delivery, the risks of neonatal near miss (NNM) and severe adverse neonatal outcomes (SNO) were significantly higher in primary CD group (aOR 1.71; 95% CI 1.17-2.51 and aOR 1.66; 95% CI 1.14-2.43), respectively. For repeated CD, the risks were borderline significant (aOR, 1.58; 95% CI, 0.98-2.56 for NNM and aOR, 1.61; 95% CI, 0.99-2.60 for SNO).

**Conclusion:**

Primary and repeated CD significantly increased the risk of SMO compared with VD. Risks of NNM and SNO were also significantly increased in women with primary CD. The risks of NNM and SNO for repeated CD trended toward a significant increase.

## 1. Introduction

Cesarean delivery (CD) is a common obstetric procedure that aims to decrease severe adverse maternal and neonatal outcomes. In 2015, the World Health Organization (WHO) however stated that “caesarean section rates higher than 10% are not associated with reductions in maternal and newborn mortality rates” [1]. In recent years, cesarean delivery has become increasingly common in both developed and developing countries. The estimated global rate of cesarean delivery between 1990 and 2014 was approximately 18.6% [2]. The absolute increase in Asia was 15.1%. In Thailand, the cesarean delivery rate was 34.1% during 2007–2008 [3].

However, there is no clear evidence that the rapid increase in the number of cesarean deliveries has led to improvements in the rates of maternal or neonatal morbidity [4]. Conversely, the rates of adverse maternal and neonatal outcomes have increased significantly in the last decade [5–7]. Women with repeated cesarean deliveries also have a greater risk of placenta abnormalities [8], such as placenta previa, [9] placental abruption, placenta accreta, uterine rupture, and unplanned hysterectomy [10] in subsequent pregnancies compared with women whose previous deliveries were vaginal. Additionally, the risk of various serious maternal morbidities has increased progressively as the number of women who have undergone cesarean deliveries has grown [11]. However, some studies found no statistically significant differences in terms of maternal morbidities, such as placenta previa and blood transfusion, between pregnant women who have had a single repeated cesarean deliveries and those who have undergone two or more [12].

In 2011, the WHO defined the term “maternal near-miss” (MNM) as “a woman who almost dies but survives a complication during pregnancy, childbirth, or within 42 days after termination of pregnancy” and suggested that MNM events and maternal deaths should be coupled to reflect severe maternal outcomes (SMO) [13]. The WHO MNM criteria help to identify issues that may lead to life-threatening conditions and can be used to monitor and improve the quality of care in maternity settings. This guide has been increasingly used as a tool to evaluate and improve the quality of maternal healthcare in many countries.

There have only been two previous studies that have explored cesarean deliveries in relation to MNM and SMO in low- and middle-income countries [14, 15], both of which indicated that women who had undergone repeated cesarean deliveries had an increased risk of MNM and SMO. However, a study from Tanzania [16] found that a history of cesarean delivery did not increase the risk of severe adverse maternal outcomes and, in fact, reduced the risk of severe adverse perinatal outcomes. Additionally, differences in terms of standards of healthcare, clinical decision making, number of women at risk, etc. may have influenced these outcomes. There has been limited data published about the risk of MNM and SMO among women with repeated CD in Thailand. The purpose of our study was to explore the risks of severe adverse maternal and neonatal outcomes in women with repeated CD and primary CD compared with those with vaginal deliveries (VD) using WHO MNM criteria.

## 2. Materials and Methods

A cross-sectional study was performed based on the medical records of pregnant women who gave birth between August 2014 and December 2016, at Khon Kaen University Faculty of Medicine's Srinagarind Hospital in Thailand. Study approval was obtained from The Khon Kaen University Ethics Committee for Human Research (HE601136).

### 2.1. Population and Sampling

Women who delivered with repeated cesarean section between August 2014 and December 2016, at Khon Kaen University Faculty of Medicine's Srinagarind Hospital in Thailand, were identified from labor room log books and were classified as first exposure group. One woman with primary CD was matched with each woman in the first exposure group by the closest date of delivery served as the second exposure group. One woman with VD was matched with each woman in the first exposure group by the closest date of delivery served as the control group.

Exclusion criteria were gestational age <22 weeks or birth weight <500 g, multifetal pregnancies, pregnancies being terminated due to fetal structural anomalies or chromosome abnormalities, and incomplete or missing data.

Medical records of these three groups of patients as well as their newborns were reviewed. Relevant data were extracted from these medical records and recorded in the pretested data collection forms.

### 2.2. Sample Size

The sample size was calculated for hypothesis testing of two independent proportions using the type 1 (*α*) error of 0.05 and power (1-*β*) of 0.8. A study by Lumbiganon et al. found the rate of severe maternal outcomes occurring in vaginal deliveries to be 1.6% [3]. We postulated that the rate of severe maternal outcomes in women with repeated CD would be 2.5 times higher. Calculations according to these values indicated a minimal sample size of 741 in each group.

### 2.3. Variables and Definitions

The primary outcomes were categorized into maternal and neonatal outcomes based on WHO MNM criteria [13]. Severe adverse maternal outcomes (SMO) were maternal death (MD) and maternal near-miss (MNM) which was morbidity with >1 organ dysfunction/life-threatening condition occurring within seven days of delivery. These included (1) cardiovascular dysfunction (defined as shock or cardiac arrest [absence of pulse/heart beat and loss of consciousness]), use of continuous vasoactive drugs, cardiopulmonary resuscitation, severe hypoperfusion (lactate >5 mmol/l or >45 mg/dl), severe acidosis (pH <7.1); (2) respiratory dysfunction was defined as acute cyanosis, gasping, severe tachypnea (respiratory rate >40 breaths per minute), severe bradypnea (respiratory rate <6 breaths per minute), intubation and ventilation not related to anaesthesia, severe hypoxemia (O2 saturation <90% for ≥60 minutes or PAO2/ FiO2 <200); (3) renal dysfunction was defined as oliguria non-responsive to fluids or diuretics, dialysis for acute renal failure, severe acute azotemia (creatinine ≥300 *μ*mol/ml or ≥3.5 mg/dl); (4) coagulation/hematological dysfunction was defined as failure to form clots, massive transfusion of blood or red cells (≥5 units), severe acute thrombocytopenia (<50,000 platelets/ml); (5) hepatic dysfunction was defined as jaundice in the presence of preeclampsia, severe acute hyperbilirubinemia (bilirubin >100 *μ*mol/l or >6.0 mg/dl); (6) neurological dysfunction was defined as prolonged unconsciousness (lasting ≥12 hours)/coma (including metabolic coma), stroke, uncontrollable fits/status epilepticus, total paralysis; and (7) uterine dysfunction was defined as uterine haemorrhage or infection leading to hysterectomy.

Neonatal near miss (NNM) was defined as Apgar score at 5 min of <7, or neonatal resuscitation, or admission to the neonatal intensive care unit occurring within seven days of delivery. Severe adverse neonatal outcomes (SNO) were a combination of neonatal death and neonatal near miss (NNM).

Potential confounding factors were fetal birth weight, maternal characteristics, and medical/obstetrical conditions. Maternal characteristics included age, level of education, pregestational BMI, and parity. Medical conditions were defined as prepregnancy diabetes mellitus, chronic hypertension, hepatitis B carrier, HIV, heart diseases, renal diseases, thyroid diseases, respiratory disease, systemic lupus erythematosus, and anemia. Obstetrical conditions were defined as pregnancy-induced hypertension, gestational diabetes mellitus.

### 2.4. Statistical Analysis

Descriptive statistics for gestational age at delivery (weeks), pregestational BMI, level of education, parity, and comorbidities during the current pregnancy were expressed as percentages. Multiple logistic regression was used to control potential confounding factors as described above. Risks of severe adverse maternal and neonatal outcomes among women with repeated CD and primary CD were compared with those with VD and were presented as adjusted odds ratio (aOR) with corresponding 95% confidence intervals (CI). All analyses were carried out using STATA 10 (Stata Corporation, College Station, TX, USA).

## 3. Results

**Figure 1:**
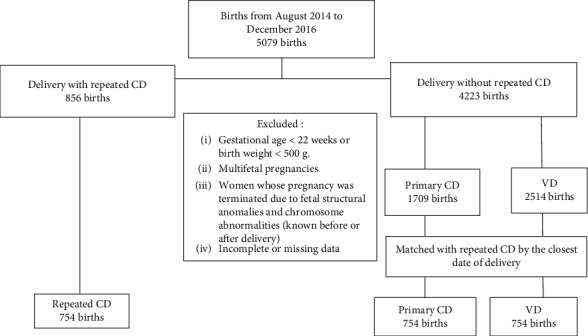
Flow diagram of participants.

Figure 1 shows the study profile. There were 856 women delivered by repeated CD between August 2014 and December 2016. We excluded 102 women with exclusion criteria, leaving 754 women with repeated CD in the study. We systematically selected 754 women with primary CD and 754 women with vaginal deliveries matched by the closet dates of delivery.

The characteristics of the study participants are presented descriptively in Table 1. Vaginal deliveries had the highest numbers of maternal age below 20 years. Bachelor's degree or higher was the most frequent level of education of women in the present study. Underweight pregestational BMI was lower in women with repeated CD, whereas overweight/obesity was higher in this group. Multiparous was the lowest in women with primary CD. The most common medical condition during pregnancy was anemia.

Table 2 shows severe adverse maternal and neonatal outcomes by mode of delivery. There were no maternal deaths. Hematological dysfunction and uterine dysfunction were the two most common MNM outcomes. Severe postpartum haemorrhage was higher in women with cesarean deliveries. There was no placenta accrete syndromes and admission to ICU in women with VD, whereas sepsis or severe systemic infection was higher in women with VD. There were five neonatal deaths; one (0.13%) in women with primary CD group, one (0.13%) in women with repeated CD, and three (0.40%) in women with VD.

Table 3 shows maternal outcomes by mode of delivery. The odds of SMO was significantly increased in women with primary CD (adjusted OR 10.59, 95% CI 1.19-94.54) and in women with repeated CD (adjusted OR 17.21, 95% CI 1.97-150.51) compared with women with VD. Severe postpartum haemorrhage was significantly increased in women with both primary and repeated CD compared with women with VD (adjusted OR 12.73, 95% CI 1.53-105.55 and 15.93, 95% CI 1.83-138.29, respectively).

Table 4 shows severe adverse neonatal outcomes (SNO) by mode of delivery. The odds of SNO was significantly increased in women with primary CD compared to women with VD (adjusted OR 1.66, 95% CI 1.14-2.43). The odds of SNO were also increased in women who with repeated CD compared with VD. However, this difference was not statistically significant, although borderline (adjusted OR 1.61 95% CI 0.99-2.60).

## 4. Discussion

Women undergoing CD carried significantly higher risks of MNM, SMO, and severe postpartum haemorrhage. For neonatal outcomes, neonates born by CD had higher rates of NNM and SNO compared with those who were born vaginally. When adjusted by potential confounding factors including maternal age, parity, birth weight, and maternal comorbidity, the higher risks of these adverse pregnancy outcomes appeared to be in the same direction for both primary and repeated CD groups.

MNM and SMO have been acknowledged as the outcomes that are strongly associated with CD [17, 18]. Mohammadi et al. [17] reported that the most significant predictor was the route of delivery. Women undergoing CD were approximately a 7.4-fold likelihood of experiencing MNM (95% CI, 3.7-15.1). A study conducted in Northern Ethiopia reported that CD has increased the odds of experiencing MNM (adjusted OR 4.6; 95% CI: 1.98, 7.61). [18] In a population-based cohort study that covered 900,108 women aged 15-44 years with singleton live births, CD was associated with a significantly increased risk of postpartum readmission (rate 2.7%; OR, 1.9; 95% CI, 1.8-1.9) compared with vaginal delivery [19]. The common postpartum complications consisted of pelvic injury/wound complication, obstetric complications, venous disorders and thromboembolism, and major puerperal infection [19]. Our findings confirm that CD is independently associated with MNM and SMO regardless of the type of CD.

Although there were no maternal deaths in this study, a recent systematic review conducted to assess the relationship between maternal death and CD in Latin America indicated an increased risk of maternal death following CD compared to vaginal delivery [20]. An elevated risk of maternal death among women undergoing CD is also noted in high-income countries. In the Netherlands, the risk of death after CD was 21.9 per 100.000 CD performed compared to that of 3.8 deaths per 100.000 vaginal births. Compared to vaginal birth, maternal mortality after CD was 3.4 times higher (95% CI, 2.4-4.8) after excluding deaths that had no association with surgery [21].

In many low- and middle-income countries, obstetric haemorrhage is the leading cause of serious maternal morbidity and mortality [22]. Obstetric haemorrhage accounted for approximately 26-46% of the MNM cases in Ethiopian women [18, 23]. In this study, we determined the association between severe postpartum haemorrhage and CD. We noted that CD increased the risk of this maternal life-threatening condition when compared to vaginal delivery. In this investigation, we also observed that among 754 women with repeated CD, there were eight cases (a prevalence of 1.06%) of placenta accrete. A recent report from the US indicated that women with previous CD had an increased risk of placenta accrete which is now considered to be an important cause of maternal death in the US [24].

A cohort study conducted in Southeast Brazil revealed higher rates of NNM and neonatal death following CD compared with vaginal delivery. Neonates born by CD carried approximately 2 times more likely to suffer from NNM (OR, 2.0; 95% CI, 1.35-2.96). However, the difference in terms of the risk of neonatal death among CD and vaginal delivery did not reach a statistically significant threshold (OR, 1.29; (95% CI 0.61-2.74) [25]. In the present study, the rates of NNM and SNO were higher among neonates born by CD compared to those who born vaginally. The risks of these two adverse outcomes were significantly higher among those in primary CD group. For repeated CD, it was found to be marginally significant when controlling for commonly applied confounding factors. However, a relatively small sample size and rarity of the occurrence of neonatal death in the present study precluded any meaningful interpretation regarding the impact of CD on the risk of neonatal death.

The strength of this study is that the adverse maternal and neonatal outcomes were measured as per the most recent approach recommended by WHO. Some limitations of this study however are worthy of note. First, this study applied retrospective data collection; some clinically important information may not be available, such as indications of CD, labor characteristics, adequacy of antenatal care, and smoking history. Second, a relative rarity of outcomes of interest has resulted in a wide confidence interval of summary measures. Third, the present study was conducted at a referral institution, which was likely to have an overrepresentation of complicated pregnancy. This might limit the extrapolation of our findings to facilities of primary or secondary healthcare settings.

## 5. Conclusions

The present study observed significant independently higher risks of MNM, SMO, and severe postpartum haemorrhage among women undergoing CD with a higher risk in repeated CD. CD has also tended to increase the risks of NNM and SNO. Based on these findings, effective interventions for reducing unnecessary CD are therefore of utmost importance to avoid adverse pregnancy outcomes that are potentially associated with CD.

## Figures and Tables

**Table 1:** Baseline characteristics of women with vaginal, primary and repeated cesarean deliveries.

Baseline characteristics	VD (*n* = 754)	Primary CD (*n* = 754)	Repeated CD (*n* = 754)
	*n* (%)	*n* (%)	*n* (%)
Maternal characteristics			
Age (year)			
<20	61 (8.09)	27 (3.58)	2 (0.27)
20-34	623 (82.63)	551 (73.08)	536 (71.09)
>35	70 (9.28)	176 (23.34)	216 (28.65)
Level of education			
<high school	87 (11.66)	74 (9.87)	69 (9.25)
High school	189 (25.34)	111 (14.80)	74 (9.92)
Vocational school	156 (20.91)	133 (17.73)	136 (18.23)
Bachelor's degree or higher	314 (42.09)	432 (57.60)	467 (62.60)
Pregestational BMI			
<18.5 (underweight)	212 (28.30)	153 (20.48)	102 (13.60)
18.5-24.9 (normal)	464 (61.95)	467 (62.52)	462 (61.60)
25-29.9 (overweight)	48 (6.41)	91 (12.18)	134 (17.87)
>30 (obese)	25 (3.34)	36 (4.82)	52 (6.93)
Multiparous	329 (43.63)	149 (19.76)	754 (100)
Medical condition			
Prepregnancy diabetes mellitus	1 (0.13)	2 (0.27)	1 (0.13)
Chronic hypertension	3 (0.40)	4 (0.53)	14 (1.86)
Hepatitis B carrier	15 (1.99)	22 (2.92)	19 (2.52)
HIV	2 (0.27)	3 (0.40)	4 (0.53)
Heart diseases	3 (0.40)	6 (0.80)	5 (0.66)
Renal diseases	2 (0.27)	3 (0.40)	3 (0.40)
Thyroid diseases	11 (1.46)	14 (1.86)	7 (0.93)
Respiratory disease	3 (0.40)	4 (0.53)	3 (0.40)
Systemic lupus erythematosus	2 (0.27)	8 (1.06)	3 (0.40)
Anemia	113 (14.99)	126 (16.71)	120 (15.92)
Obstetric condition			
Pregnancy induced hypertension	19 (2.52)	41 (5.44)	22 (2.92)
Gestational diabetes mellitus	38 (5.04)	76 (10.08)	72 (9.55)
Neonatal characteristics			
Birthweight (g)			
<2,500	69 (9.16)	71 (9.43)	57 (7.56)
2,500-4,000	677 (89.91)	654 (86.85)	676 (89.66)
>4,000	7 (0.93)	28 (3.72)	21 (2.79)
Gestational age at delivery (week)			
Preterm (<37)	70 (9.28)	63 (8.36)	86 (11.41)
Term (37-41)	683 (90.58)	690 (91.51)	668 (88.59)
Postterm (>42)	1 (0.13)	1 (0.13)	0 (0.00)

VD: vaginal delivery; CD: cesarean delivery.

**Table 2:** Maternal and neonatal outcomes of women with vaginal, primary and repeated cesarean deliveries.

	VD (*n* = 754)	Primary CD (*n* = 754)	Repeated CD (*n* = 754)
	*n* (%)	*n* (%)	*n* (%)
Maternal outcome			
Maternal vital status			
(i) Maternal death	0 (0.00)	0 (0.00)	0 (0.00)
Severe maternal complications			
(i) Severe postpartum haemorrhage	1 (0.13)	10 (1.33)	16 (2.12)
(ii) Sepsis or severe systemic infection	13 (1.72)	4 (0.53)	3 (0.40)
(iii) Placental previa	0 (0.00)	20 (2.65)	21 (2.79)
(iv) Placenta accrete syndromes	0 (0.00)	2 (0.27)	8 (1.06)
(v) Placenta abruption	1 (0.13)	4 (0.53)	0 (0.00)
(vi) Ruptured uterus	0 (0.00)	0 (0.00)	0 (0.00)
Critical interventions			
(i) Admission to intensive care unit	0 (0.00)	12 (1.59)	16 (2.12)
(ii) Interventional radiology	0 (0.00)	0 (0.00)	0 (0.00)
(iii) Laparotomy (includes hysterectomy, excludes caesarean section)	0 (0.00)	2 (0.27)	4 (0.53)
(v) Massive blood transfusion	0 (0.00)	4 (0.53)	8 (1.06)
Maternal near-miss			
(i) Cardiovascular dysfunction	0 (0.00)	2 (0.27)	1 (0.13)
(ii) Respiratory dysfunction	0 (0.00)	5 (0.66)	7 (0.93)
(iii) Renal dysfunction	0 (0.00)	0 (0.00)	2 (0.27)
(iv) Hepatic dysfunction	0 (0.00)	0 (0.00)	0 (0.00)
(v) Neurological dysfunction	0 (0.00)	1 (0.13)	0 (0.00)
(vi) Hematological dysfunction	1 (0.13)	4 (0.53)	8 (1.06)
(vii) Uterine dysfunction	0 (0.00)	2 (0.27)	10 (1.33)
Neonatal outcomes			
(i) Apgar score at 5 minutes <7	5 (0.66)	14 (1.86)	11 (1.46)
(ii) Neonatal resuscitation	59 (7.82)	99 (13.13)	69 (9.15)
(iii) NICU admission	15 (1.99)	30 (3.98)	23 (3.05)
(iv) Intrapartum deaths	3 (0.40)	0 (0.00)	1 (0.13)
(v) Early neonatal death	0 (0.00)	1 (0.13)	0 (0.00)

CD: cesarean delivery; VD: vaginal delivery.

**Table 3:** Maternal outcomes by mode of delivery.

Maternal outcome	VD	Primary CD		Repeated CD	
	Reference group	Crude OR [95% CI]	aOR [95% CI]	Crude OR [95% CI]	aOR [95% CI]
Maternal death	1	—	—	—	—
Maternal near miss (MNM)	1	9.10 (1.15-71.98)	10.59 (1.19-94.54)	16.32 (2.16-123.41)	17.21 (1.97-150.51)
Severe maternal outcomes (SMO)	1	9.10 (1.15-71.98)	10.59 (1.19-94.54)	16.32 (2.16-123.41)	17.21 (1.97-150.51)
Severe postpartum haemorrhage	1	10.12 (1.29-79.26)	12.73 (1.53-105.55)	16.33 (2.16-123.41)	15.93 (1.83-138.29)

Adjusted by maternal age, level of education, parity, pregestational BMI, medical and obstetrical conditions, and birth weight (g). Women with vaginal deliveries are the reference. CD: cesarean delivery; VD: vaginal delivery.

**Table 4:** Neonatal outcomes by mode of delivery.

Neonatal outcomes	VD	Primary CD		Repeated CD	
	Reference group	Crude OR [95% CI]	aOR [95% CI]	Crude OR [95% CI]	aOR [95% CI]
Neonatal deaths	1	0.33 (0.03-3.20)	0.34 (0.01-6.73)	0.33 (0.03-3.20)	Not estimable
Neonatal near miss (NNM)	1	1.80 (1.28-2.53)	1.71 (1.17-2.51)	1.21 (0.84-1.73)	1.58 (0.98-2.56)
Severe neonatal outcomes (SNO)	1	1.73 (1.24-2.41)	1.66 (1.14-2.43)	1.16 (0.81-1.66)	1.61 (0.99-2.60)
Apgar score at 5 minutes <7	1	2.83 (1.02-7.91)	3.74 (1.06-13.17)	2.21 (0.77-6.41)	7.45 (1.28-43.46)
Neonatal resuscitation	1	1.78 (1.27-2.50)	1.72 (1.17-2.53)	1.19 (0.83-1.71)	1.56 (0.96-2.52)
Preterm birth (<37 weeks)	1	0.89 (0.62-1.27)	0.88 (0.55-1.41)	1.26 (0.90-1.76)	1.09 (0.68-1.74)
Low birth weight (<2500 g)	1	1.03 (0.73-1.46)	0.90 (0.60-1.35)	0.81 (0.56-1.17)	0.93 (0.58-1.49)

Adjusted by maternal age, level of education, parity, pregestational BMI, medical and obstetrical conditions and birth weight (g). Women with vaginal delivery is the reference. CD: cesarean delivery; VD: vaginal delivery.

## Data Availability

The data used to support the findings of this study are available from the corresponding author upon request.
